# Severe Infective Endocarditis Caused by *Bartonella rochalimae*

**DOI:** 10.3201/eid3002.230929

**Published:** 2024-02

**Authors:** Edward C. Traver, Kapil Saharia, Paul Luethy, Anthony Amoroso

**Affiliations:** University of Maryland School of Medicine, Baltimore, Maryland, USA

**Keywords:** Bartonella rochalimae, endocarditis, next-generation sequencing, bacteria, United States, Guatemala

## Abstract

A 22-year-old man from Guatemala sought care for subacute endocarditis and mycotic brain aneurysm after living in good health in the United States for 15 months. *Bartonella rochalimae*, a recently described human and canine pathogen, was identified by plasma microbial cell-free DNA testing. The source of infection is unknown.

A 22-year-old man with a history of an unrepaired congenital ventricular septal defect (VSD) experienced 3 months of progressive dyspnea on exertion, weight loss, and fatigue and 2 weeks of debilitating weakness. He had been born in Guatemala, where he worked in construction; he had had contact with goats, horses, cattle, and chickens but reported no contact with dogs or cats. Eighteen months before he sought care, he had migrated to the mid-Atlantic region of the United States, where he lived with his uncle and a few other adults in a suburban town. He continued to work in construction, did not use illicit drugs, and had 1 female sexual partner. Six months after he arrived, his uncle took in a stray dog.

The patient was afebrile, hypotensive, bradycardic, and thin. A systolic ejection murmur and a fourth heart sound were present. He had right upper quadrant abdominal tenderness and digital clubbing. Laboratory studies revealed anemia, unremarkable creatinine levels, and elevated liver enzymes (Table). Results of 3 sets of bacterial blood cultures and 1 set of fungal blood cultures were negative. Transthoracic and transesophageal echocardiograms demonstrated a VSD with bidirectional shunting and a mobile mitral valve echodensity. We initiated treatment for culture-negative infective endocarditis (Figure). A computed tomography angiogram of the brain, performed on day 2, revealed a 2–3 mm mycotic aneurysm in the right frontal middle cerebral artery; it appeared smaller by digital subtraction angiography on day 7.

**Table:** Laboratory results for patient with infective endocarditis caused by *Bartonella rochalimae*, United States*

Test	Result	Reference range
Leukocytes, K/μL	4.8	4.5–11.0
Hemoglobin, g/dL	10.9	12.6–17.4
Platelets, K/μL	168	153–367
Creatinine, mg/dL	0.77	0.66–1.25
AST, units/L	**84 **	17–59
ALT, units/L	**58 **	0–49
Alkaline phosphatase, units/L	109	38–126
Total bilirubin, mg/dL	0.6	0.3–1.2
CRP, mg/dL	**3.7 **	<1.0
ESR, mm/h	**81 **	0–15
4th-generation HIV antigen and antibody test	Nonreactive	Nonreactive
*Coxiella burnetii* Phase 2 IgM	**1:32**	Negative
*C. burnetii* Phase 2 IgG	**1:128**	Negative
*C. burnetii* Phase 1 IgM	Negative	Negative
*C. burnetii* Phase 1 IgG	**1:16**	Negative
*Brucella* antibody agglutination	<1:20	<1:20
*Chlamydia pneumoniae* IgM	<1:20	<1:20
*C. pneumoniae* IgG	**1:512**	<1:64
*C. trachomatis* IgM	<1:20	<1:20
*C. trachomatis* IgG	**1:128**	<1:64
*C. psittaci* IgM	<1:20	<1:20
*C. psittaci* IgG	**1:512**	<1:64
*Bartonella henselae* IgG	**>1:1024**	Unknown
*B. henselae* IgM	**1:64**	Unknown
*B. quintana* IgG	**>1:1024**	Unknown
*B. quintana* IgM	<1:16	Unknown

*Bold text indicates abnormal values. ALT, alanine transaminase; AST, aspartate transferase; CRP, C-reactive protein; ESR, erythrocyte sedimentation rate.

**Figure:**
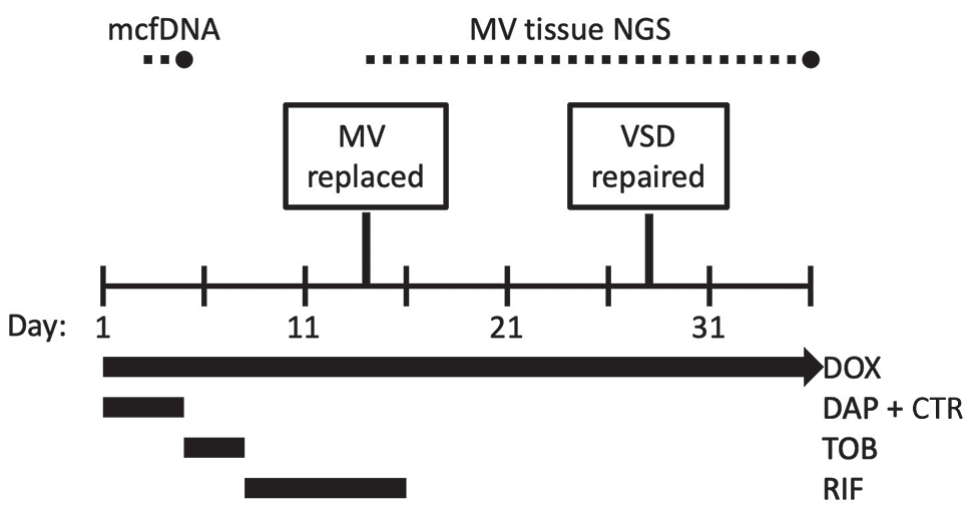
Diagnostics and treatments for *Bartonella rochalimae* endocarditis during a 36-day hospitalization. Dotted lines indicate the time between specimen collection and test result for mcfDNA and MV tissue NGS tests. CTR, ceftriaxone; DAP, daptomycin; DOX, doxycycline; mcfDNA, microbial cell-free DNA; MV, mitral valve; NGS, next-generation sequencing; RIF, rifampin; TOB, tobramycin; VSD, ventricular septal defect.

On hospital day 2, we sent a plasma microbial cell-free DNA (mcfDNA) test to Karius (Redwood City, CA, USA). The result on day 4 was positive for *Bartonella rochalimae* DNA (47,501 molecules/μL; reference <10 molecules/μL). We changed the antimicrobial drug regimen to target *Bartonella* endocarditis and adjusted it over the hospital stay to avoid toxicities and interactions (Figure). We performed serologic tests for *Bartonella*, *Brucella*, *Chlamydia*, and *Coxiella* species (Table).

Thirteen days after admission, the mitral valve was surgically replaced; both valve leaflets demonstrated chronic-appearing vegetations, and the anterior leaflet was perforated. Results for bacterial culture of the valve tissue were negative; histopathology was not performed. Mitral valve tissue 16S ribosomal RNA amplicon-based next-generation sequencing (NGS) was performed by the University of Washington Department of Laboratory Medicine Molecular Diagnosis Microbiology Section (Renton, WA, USA). After mitral valve replacement, the left-to-right shunt increased, and the patient underwent a second sternotomy and open VSD repair. The amplicon-based NGS test of the mitral valve tissue result, received on hospital day 36, was positive for *B. rochalimae*. The patient was discharged and instructed to complete an additional 3-month course of oral doxycycline. However, he moved out of the region 2 months later, so no follow-up visits occurred.

*B. rochalimae* was first isolated from a person traveling from the United States to Peru and demonstrated to be the cause of her febrile illness in 2007 (*1*). It has since been detected in 1 other human (*2*). We report a case of *B. rochalimae* human infective endocarditis in a man with an unrepaired congenital heart disease, diagnosed by plasma mcfDNA and confirmed to the species level by NGS of the endocardial tissue. Results of serologic tests for other *Bartonella* species and *Coxiella* and *Chlamydia* species may be elevated because of co-exposure or cross-reactivity (*3*,*4*). *B. rochalimae* has not been detected in other clinical samples by the Karius test (L. Dyner, Karius, Inc., pers. comm., email, 2023 Feb 17) and was not detected among 168 *Bartonella* spp. identified to the species level by 16S PCR at the University of Washington during 2003–2021 (*5*).

A published case of human disease occurred in a traveler who had been exposed to arthropods in Peru; a closely related, possibly identical, species named AF415211, was collected from a flea in Peru in 1998 (*1*,*6*). It is unclear if *B. rochalimae* exists in Guatemala, where the patient we report lived, or if the infection was subclinical for the 15 months he was living in the United States in good health. *B. rochalimae* has caused infective endocarditis in dogs in the United States and Europe and has been detected by PCR in the fleas or blood of dogs throughout the world (*6*–*8*). Dogs, foxes, and coyotes may be natural reservoirs. Our patient did report exposure to a dog, but only after arriving in the mid-Atlantic region of the United States. The location and vector of this patient’s infection require further investigation, but our findings illustrate that mcfDNA may be useful to identify of new and emerging pathogens.
